# Supplementary material for: Venomix: a simple bioinformatic pipeline for identifying and characterizing toxin gene candidates from transcriptomic data
Source: PeerJ. 2018 Jul 31;6:e5361. doi: 10.7717/peerj.5361 (PMC6074769; doi:10.7717/peerj.5361)
Supplement: Supplemental Information 6 [file peerj-06-5361-s006.gz › FinalOutput_1E-6/La1-like_protein_13_1/finaltree.pdf]

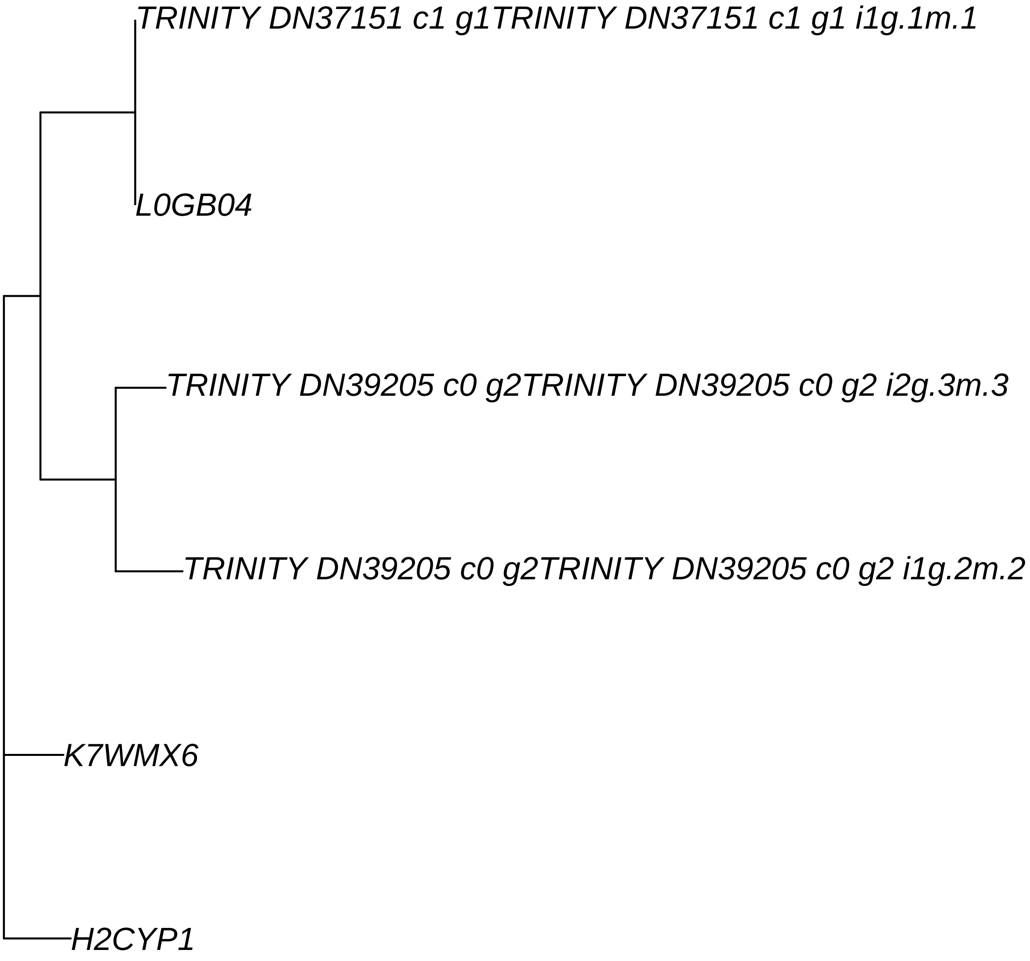
TRINITY DN37151 c1 g1TRINITY DN37151 c1 g1 i1g.1m.1
L0GB04
TRINITY DN39205 c0 g2TRINITY DN39205 c0 g2 i2g.3m.3
TRINITY DN39205 c0 g2TRINITY DN39205 c0 g2 i1g.2m.2
K7WMX6
H2CYP1
